# Action and Interaction between Retinoic Acid Signaling and Blood–Testis Barrier Function in the Spermatogenesis Cycle

**DOI:** 10.3390/cells11030352

**Published:** 2022-01-21

**Authors:** Yu Zhou, Yunyan Wang

**Affiliations:** 1 Department of Child Rehabilitation Division, Huai’an Maternal and Child Care Hospital, Huai’an 223002, China; zhouy178@stu.cqmu.edu.cn; 2 Department of Urology, The Affiliated Huai’an No.1 People’s Hospital of Nanjing Medical University, Huai’an 223300, China

**Keywords:** retinoic acid, spermatogenesis, blood–testis barrier, meiosis, male infertility

## Abstract

Spermatogenesis is a complex process occurring in mammalian testes, and constant sperm production depends on the exact regulation of the microenvironment in the testes. Many studies have indicated the crucial role of blood–testis barrier (BTB) junctions and retinoic acid (RA) signaling in the spermatogenesis process. The BTB consists of junctions between adjacent Sertoli cells, comprised mainly of tight junctions and gap junctions. In vitamin A-deficient mice, halted spermatogenesis could be rebooted by RA or vitamin A administration, indicating that RA is absolutely required for spermatogenesis. Accordingly, this manuscript will review and discuss how RA and the BTB regulate spermatogenesis and the interaction between RA signaling and BTB function.

## 1. Introduction

**Figure 1:**
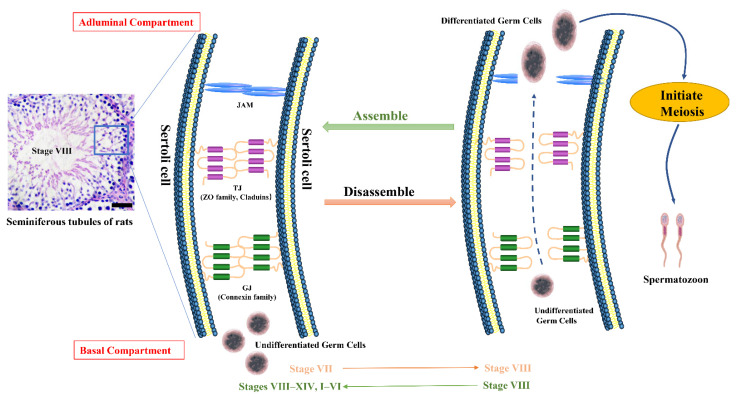
The blood–testis barrier functions in germ cell differentiation. The section with blue boxed area is a normal testis in adult rats with hematoxylin-eosin staining. The BTB is mainly composed of TJs and GJs of adjacent Sertoli cells. ZO-1, Occludin and Claudin-11 are major genes for TJ function, and Connexin-43 is the crucial gene for GJ function. The junctions divide the BTB into adluminal and basal compartments. During stages VII–VIII of the spermatogenic cycle, the “old” BTB is disassembled to allow germ cell differentiation in the adluminal compartment and then enters the meiosis process. The “old” BTB will be reassembled after stage VIII to maintain homeostasis in the testis. BTB, blood–testis barrier. TJ, tight junction. GJ, gap junction. Scale bar: 200 μm.

Spermatogenesis is a highly organized and complex process. Spermatogonial stem cells (SSCs) differentiate into spermatozoa in a cyclical process, and this seminiferous epithelial cycle can produce sperm continuously [1]. In the mouse testis, 12 germ cell associations were identified that represent seminiferous epithelial stages I–XII, while there were 14 seminiferous epithelial stages (I–XIV) in the rat testis. In humans, I–VI stages are detected in testis. The germ cells are arranged along the longitudinal axis of the epithelial tubule and become stage-dependent to produce spermatozoa continuously (Figure 1).

As early as 3–4 days after birth, undifferentiated (A_undiff_) and differentiated (A_diff_) spermatogonia cells could be detected. A part of the A_undiff_ population is responsible for keeping the SSC pool at a steady-state for spermatogenesis, and the remaining neonatal spermatogonia become progenitors or differentiating spermatogonia [2,3]. Individual spermatogonia cells are referred to as A single (A_s_) spermatogonia. A_s_ spermatogonia can divide into paired A (A_p_) spermatogonia cells and then form a chain of 4–32 aligned (A_al_) spermatogonia. A_p_ and A_al_ spermatogonia are recognized as undifferentiated spermatogonia that retain stem cell properties [4]. Undifferentiated spermatogonial cells could differentiate into type A1 spermatogonia (A1), which marks the initiation of meiosis. A1 spermatogonia transform to A2, A3, A4, intermediate, and B spermatogonia and finally become preleptotene spermatocytes (Figure 1) [5,6].

Spermatogenesis progression absolutely requires a proper microenvironment formed by somatic cells. Sertoli cells are unique somatic cells that can form the blood–testis barrier (BTB) to maintain homeostasis in testes [7]. The BTB is one of the tightest blood–tissue barriers because it consists of tight junctions (TJs), gap junctions (GJs), demosomes, and ectoplasmics [8]. The BTB can limit the diffusion of substances and prevent germ cells from immune cells in the circulatory and peripheral immune systems [9,10]. The disruption of BTB junctions will cause male infertility because germ cells will fail to cross from the basal to the adluminal compartment and will be stalled in an undifferentiated state [11]. The BTB can be regulated by various signaling pathways, and retinoic acid (RA) is indispensable for BTB formation and function.

In vitamin A-deficient (VAD) mice, spermatogenesis is inhibited, and the administration of RA to VAD mice can promote synchronized spermatogenesis [12,13]. When retinoic acid receptor (RAR) alpha is knocked out in Sertoli cells, the seminiferous epithelium degenerates, and only premiotic germ cells and undifferentiated spermatogonia are left in the spermatogenesis cycle [14]. We, therefore, clarified the effect of RA on the spermatogenesis process. The discussion of RA synthesis and RA-related molecular functions is mainly based on rats in vivo, mouse models, and cell culture data in vitro.

Many articles have reviewed the role of RA or BTB junctions in the spermatogenesis process, while fewer have noticed the interaction between RA and BTB functions. Thus, this review will review the effect of RA and BTB junctions in normal spermatogenesis and particularly summarize the interaction between RA signaling and BTB functions.

## 2. The Role of BTB on Spermatogenesis

Sertoli cell-Sertoli cell interactions form a complex network with direct junctions, that also allow the transmission of molecular signals. The BTB rigidly compartmentalizes the seminiferous epithelium into basal and apical compartments [15]. Spermatogonia and preleptotene spermatocytes reside in the basal compartment. Pachytene spermatocytes, round spermatids, and elongated spermatids reside in the apical domain. The major function of the BTB is to provide an immune-privileged microenvironment in the testis to guard the normal spermatogenesis process [15]. However, the BTB is not permanent; it needs to “open” to ensure preleptotene spermatocyte transfer into the adluminal compartment and then initiate meiosis [16]. This transformation process requires junction disassembly in stages VII–VIII of the spermatogenic cycle, and new junctions must be subsequently assembled to maintain BTB function [17]. TJ and GJ are known as the main ingredients of BTB. The crucial proteins in the junctions of the BTB are listed in Table 1.

### 2.1. The Tight Junctions

**Figure 2:**
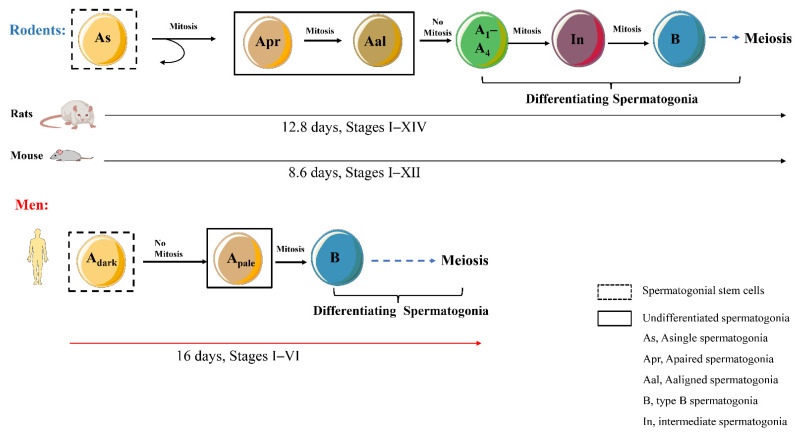
The cell division and seminiferous epithelial cycle of spermatogenesis in rodents and men. In testes of rodents, an As spermatogonia can produce two Apr cells spermatogonia through mitosis, then divide into Aal spermatogonia. As spermatogonia is referred as spermatogonial stem cells, and Apr and Aal spermatogonia are considered as undifferentiated cells. Aal spermatogonia transform into A1 spermatogonia without mitosis. A1 spermatogonia divide into Intermediate and B spermatogonia, subsequently, meiosis begins. It takes 12.8 days to complete a seminiferous epithelial cycle through 14 stages in rats, and 12 stages could be finished with 8.6 days in mouse testes seminiferous tubule. Adark spermatogonia are replicated to produce Apale spermatogonia, and then form type B spermatogonia which enter meiosis process in men. A cycle with 6 stages of the seminiferous epithelial is completed in 16 days in men.

Many proteins have been detected in TJs of Sertoli cells, mainly including claudin family proteins, junctional adhesion molecule (JAM) family proteins, and adenovirus receptor (CAR) [18] proteins (Figure 2). Claudin family proteins and junctional adhesion molecule family proteins are major members of TJs. Claudin family proteins are transmembrane proteins essential for TJ formation. In mammalian testes, a total of 24 claudins have been identified. In undescended testes of rats, claudin-11 is localized as vertical extensions at 4 weeks after birth, while it appears as parallel extensions at 5 weeks after birth, and abundant germs cells undergo apoptosis in the adluminal compartment [19]. The zonula occludens family proteins (ZO-1, -2, -3) and tethers TJs to the actin cytoskeleton [20]. It has been reported that ZO-1 and occludin share the same domain for TJ function [21]. However, in occludin-deficient intestinal epithelial cells, ZO-1 could still be recruited in TJs [21]. It was deduced that occludin is important for TJs but not indispensable.

After CAR knockdown in Sertoli cells in vitro with RNAi, the TJ barrier is disrupted and causes an increase in the expression of occludin in endocytosis [22]. In contrast, the localization of claudin-3, occludin, JAM, and ZO-1 is normal in CAR knockout mice, and the BTB is intact [23]. Thus, CAR may have no direct role in testicular development and BTB formation. We have reported that proteins of TJs (claudin-11, occludin, ZO-1) were significantly decreased in congenital cryptorchidism rats [24].

Thus, TJs are the most crucial “gate” junction for maintaining BTB functions and make the BTB one of the tightest blood–tissue barriers in mammals. Future studies should investigate the detailed interaction mechanism between TJ proteins in the BTB formation process. In addition, TJ functional changes should also be noticed in some abnormal situations, such as cryptorchidism and endocrine-disrupting chemical damage to the testis. This may be beneficial for learning about the stage variation of Sertoli functions during the spermatogenic cycle and targeted drug development for male contraception by the regulation of TJ functions.

### 2.2. The Gap Junctions

GJs are another vital junction for BTB formation, regulation, and dynamics. Unlike the “structural role” of TJs in the BTB, GJs can transmit signals to regulate the cyclic restructuring of the BTB and allow spermatogonial differentiation within the stem cell niche [25,26]. The “communication role” of GJs is achieved with one channel between adjacent Sertoli cells, as well as Sertoli cells and germ cells. Peptides, second messengers, and nucleotides could pass through the GJ channel to facilitate function.

There are twenty different connexin (CX) genes for gap junctions in mice, and CX43 (Gja1) is currently considered the most predominant GJ protein for the BTB in different species [27,28,29]. We will review the GJ function in the BTB by studying CX43. Global CX43KO mice will die at birth due to cardiac malformation [30], so a Sertoli cell-specific CX43 knockout model was developed in recent years [31]. Adult homozygous male SCCx43KO−/− mice predominantly show spermatogenesis inhibition and Sertoli cells only in the seminiferous tubules, while they could still form a functional BTB during puberty in these mice [26]. This shows that GJs are not indispensable for maintaining the physical barrier in testes.

However, after the disruption of TJs and actin microfilaments by perfluorooctanesulfonate (an environmental toxin), overexpression of CX43 could re-establish the Sertoli TJ barrier and actin microfilament organization plus redistribute TJ and basal ES proteins to the Sertoli cell–cell interface [32]. CX43 was knocked down in primary cultures of Sertoli cells by RNAi. Sertoli cells also showed a significant delay in the TJ permeability barrier, and immunofluorescence indicated the expression of TJ junction proteins (ZO-1, occludin, N-cadherin) was reduced [25]. The levels of occludin, N-cadherin, and beta-catenin were enhanced, whereas ZO-1 was reduced in conditional SSCx43 KO mice [33]. Collectively, CX43 may not be necessary for the maintenance of BTB integrity but could regulate the function of TJs and other junctions of the BTB by signal transduction with GJ channels.

Next, the effect of CX43 on other cell constituents in the testis should be investigated and not be limited to Sertoli cells, such as germ cells. In one recent study, although CX43 conditional KO mice in premeiotic and meiotic germ cells were reported [34], the detailed function of CX43-related GJ channels in spermatogenic cells of different stages needs further study. In addition, it is interesting that CX43 knockout mice have normal levels of Leydig cells, which may be interpreted as CX36 and CX45 compensating for CX43 loss in Leydig cells. The lumen of the seminiferous tubule was collapsed in CX46 knock mice [35], but no further research has been reported. Therefore, other types of connexin family proteins may also play potential functional roles in the testis. Furthermore, changes in GJ proteins are also worth discussing in some reproductive diseases. For example, the expression of CX43 is significantly decreased, caused by BTB disruption and spermatogenesis inhibition in testis tissue from COVID-19 cases [36].

## 3. The Role of RA Signaling in the Cycle of Spermatogenesis

The first report about RA and spermatogenesis was carried out in 1925 [37], and subsequent studies found that most germ cells were arrested at the undifferentiated stage in vitamin A-deficient (VAD) mice [38,39]. This shows that the transition of A_al_ into A_1_ spermatogonia requires RA activation. In VAD rats or inhibition of local RA production with WIN18446, exogenous RA injection could drive the undifferentiated population into the differentiation pathway or cause synchronous spermatogenesis in testes [40,41,42]. Thus, the regulation of RA concentration is crucial to the regulation of spermatogenesis.

**Figure 3:**
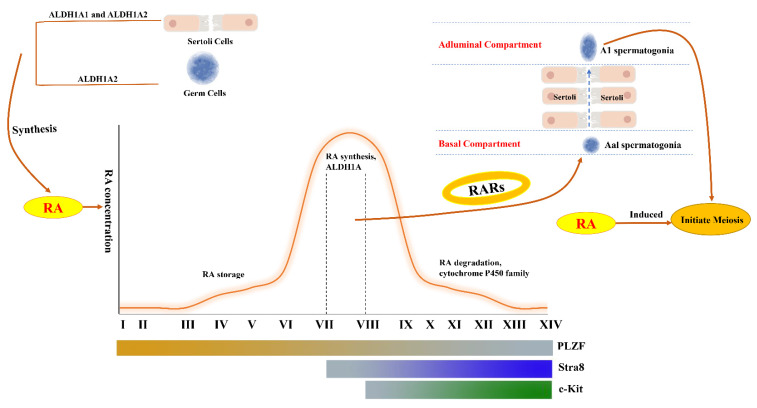
The role of retinoic acid pulse in the cycle of the seminiferous epithelium. The production of RA is mainly from Sertoli cells and germ cells in the testis after birth, and this process is mainly catalyzed by ALDH1A1 and ALDH1A2. The concentration of RA is stored in the seminiferous epithelium during stages I to VI, and rises to a peak during stages VII–VIII, and is then gradually degraded by CYP26 enzymes. The transition of Aal spermatogonia to A1 spermatogonia also occurs during stages VII–VIII. The Aal-A1 transition and meiosis process could be induced by RA administration. RA binds to RAR and can activate some downstream genes. The expression patterns of PLZF, Stra8, and c-Kit are depicted with gradual change lines.

The concentration of RA is a precise dynamic modulation process. The synthesis of RA from vitamin A requires two oxidation steps. The first step is catalyzed by alcohol dehydrogenases (ADHs) or microsomal retinol dehydrogenases (RDHs), while the second reaction requires the aldehyde dehydrogenase 1A (ALDH1A1, ALDH1A2, and ALDH1A3) family [43,44,45]. The first oxidation process is the rate-limiting step. In general, there are two outcomes after RA enters the testis: its biological function of binding to the retinoic acid receptor (RAR) or its degradation by cytochrome P450 family enzymes (CYP26A1, CYP26B1, and CYP26C1) [46,47] (Figure 3). In adult testes, ALDH1A1 and ALDH1A2 can be detected in Sertoli cells, and ALDH1A2 is detected in spermatogonia [48,49,50]. This may indicate that the endogenous biosynthesis of RA may be from Sertoli cells and germ cells in the testis after birth.

In addition, the concentration of RA changes periodically in the internal environment of the testis [42]. Stra8 is required for meiotic initiation and is stimulated directly by RA in the testis. The level of Stra8 is low in stages II–VI, subsequently increases during the A_al_-A1 transition stage (stage VII–VIII), and then remains at a high level after stages IX [51]. The expression of Stra8 could reflect the change rule of RA in mature testes.

The concentration of RA is also low in stages II–VI and reaches the peak level at stages VIII-IX [52,53,54]. After spermatogonia differentiation, the level of CYP26A1 reaches its highest level at stages VIII-XI for the degradation of RA [55] (Figure 2). This shows the concentration change of RA is spatiotemporal synchronization with spermatogenesis. The concentration of RA could regulate RA production through a feedback effect in testes, however needs to be proved by further experiments.

Hence, the pulsatile change in RA in the testis could regulate the spermatogenesis cycle in a precise and ordered path, especially the transition of A_al_-A1 is RA-depended in testes. It has been reported that the metabolism of RA is abnormal in congenital cryptorchidism testes [56]. However, the underlying mechanism of the RA-induced spermatogenesis process is still unclear, and further research should investigate the change in RA concentration in male reproductive diseases, such as in varicocele and asthenospermia patients.

## 4. The Interactions between RA and BTB

RA binds to RAR and forms a heterodimer with retinoic X receptors (RXRs) to stimulate the expression of RA-responsive genes directly or indirectly, such as Stra8, SCP3, and PLZF [44,57] (Figure 2). RARα and RARβ are also expressed in Sertoli cells [58,59]. During stages VIII–IX of the seminiferous epithelium, the BTB disassembles to complete the A_al_–A1 transition, and the level of RA also increases to a peak. Thus, there must be some interrelationships between RA signaling and the BTB.

Few studies of RA signaling and the BTB were reported until 2006 Vernet et al. deduced that RARs could act on Sertoli cells when RARα was ablated in adult mouse Sertoli cells, the expression of Stra8 was delayed, indicating that RARα is Sertoli cell-autonomous in the testis [60]. The BTB is disrupted during stages VII–XII in a negative form of RARα in Sertoli cells, and the gene expression of tight junctions is decreased [61]. In addition, blocking RA signaling in Leydig cells could cause BTB damage, decreased testosterone, and loss of advanced germ cells [62]. These studies indicate that RA signaling acts as a basic regulator of BTB disassembly and reassembly during stages VII–XII for spermatogonial transformation.

Furthermore, the ALDH1A1 enzyme in Sertoli cells is detected in stages I–VIII, while ALDH1A2 is expressed in stages VII–XII, indicating that RA production in Sertoli cells may occur earlier than in germ cells for spermatogonial differentiation [52,63]. The first round of spermatogonial differentiation is arrested when ALDH1A1 is deleted in Sertoli cells, and this process could be rescued by RA injection, which suggests that the production of RA from Sertoli cells is crucial for A_al_–A1 conversion [58]. This indicates that RA production and storage from Sertoli cells is indispensable for A_al_–A1 differentiation and meiotic initiation by binding to RARs based on the above information.

Although endogenous RA synthesis is blocked in mice with WIN18,446 treatment, synchronous spermatogenesis could be induced after RA injection in adults [64]. In addition, it is interesting that a signal injection of RA could be maintained for more than one week and induce some spermatogonial cells to undergo differentiation immediately and then initiate meiosis to restore BTB function [42,50]. Studies have shown that RA injection only drives a cohort of undifferentiated spermatogonia into A1 spermatogonia [64,65], and these A1-type spermatogonia struggle to enter meiosis [66]. However, we still do not know whether all germ cells are sensitive to exogenous RA exposure or RA production from Sertoli cells, or whether some germ cells retain the characteristics of stem cells.

Subsequent studies should discuss the detailed mechanism of the specific role of RA signaling in Sertoli cell functions and the interaction between RA production in testes and spermatogenesis. Single-cell sequencing may commendably investigate whether certain subpopulations or all spermatogonia could be induced to differentiate by RA administration, but no related study was reported in the database. Additionally, the concentration of RA is precisely modulated in the peripheral blood intake process and local production in the testis because of the permeability function of the BTB, while RA may be transferred to the testis when the BTB is destroyed by diseases. Furthermore, whether RA supplementation has a therapeutic effect on male infertility diseases needs to be confirmed in further studies.

## 5. Conclusions and Future Perspectives

Previous studies have provided much evidence about the critical role of RA and BTB in the spermatogenesis process, especially for spermatogonia differentiation and meiosis initiation. This review has summarized the functions of periodic assembly of the BTB in the seminiferous epithelium cycle and the production and pulsatile change of RA in crucial steps of the spermatogenesis process. In particular, we summarized the interaction between RA signaling and BTB functions in the present article.

Further works should employ novel research methods to improve the questionable aspects of RA and BTB in spermatogenesis. For example, global knockout of CX43 will cause mouse death, and a novel organotypic culture method could mimic BTB formation in vitro with fresh, frozen, and thawed testes, which showed almost concurrent changes in vivo [67]. This method may provide suitable methods for investigating detailed and accurate information about CX43 functions in the spermatogenic microenvironment. In addition, most studies of RA and spermatogenesis are based on animal models; only one group reported that the level of RARα was decreased in varicocele samples, and RA treatment could reprogram sperm metabolism close to capacitation status [68,69]. Future studies should also investigate the role of RA signaling in some reproductive diseases to address the treatment difficulties of male infertility in the clinic. Collectively, we would like this review to develop an understanding of how RA and the BTB contribute to spermatogenesis.

## Figures and Tables

**Table 1:** The main proteins of blood–testis junctions in rodent testes.

Junction Type	Main Proteins
Tight Junction	
Membrane proteins	Occludin, Claudin-1, -3, -4, -5, -7, -8, -11, JAM, CAR
Adaptors	ZO-1, -2, -3, Vinculin
Scaffolding proteins	Actin, Collagen IV
Gap Junction	
Membrane proteins	Connexin-43, -33, -26, -45, -46, -57
Adaptors	β-catenin,
Scaffolding proteins	Actin
Demosomes	
Membrane proteins	Desmoglein-2, Desmocollin-2, -3
Adaptors	γ-catenin, Desmoplakin
Scaffolding proteins	Vimentin
Ectoplasmic	
Membrane proteins	N-cadherin, E-cadherin, CAR
Adaptors	α-catenin, β-catenin, γ-Catenin
Scaffolding proteins	Actin

JAM, junctional adhesion molecule; CAR, coxsackievirus and adenovirus receptor; MMP, matrix metallopoteinase, ZO, zonula occludens.
